# Common effects of attractive and repulsive signaling: Further analysis of Mical-mediated F-actin disassembly and regulation by Abl

**DOI:** 10.1080/19420889.2017.1405197

**Published:** 2018-01-12

**Authors:** Jimok Yoon, Jonathan R. Terman

**Affiliations:** Departments of Neuroscience and Pharmacology, Harold C. Simmons Comprehensive Cancer Center, The University of Texas Southwestern Medical Center, Dallas, Texas, USA

**Keywords:** Attraction, Cytoskeleton, Guidance Cues, Kinase, Oxidation, Post-translational Modification, Phosphorylation, Repulsion, Semaphorin, Plexin

## Abstract

To change their size, shape, and connectivity, cells require actin and tubulin proteins to assemble together into long polymers – and numerous extracellular stimuli have now been identified that alter the assembly and organization of these cytoskeletal structures. Yet, there remains a lack of defined signaling pathways from the cell surface to the cytoskeleton for many of these extracellular signals, and so we still know little of how they exert their precise structural effects. These extracellular cues may be soluble or substrate-bound and have historically been classified into two independently acting and antagonistic groups: growth-promoting/attractants (inducing turning toward the source of the factor/positive chemotropism) or growth-preventing/repellents (turning away from the source of the factor/negative chemotropism). Paradoxically, our recent results directly link the action of growth factors/chemoattractants and their signaling pathways to the promotion of the disassembly of the F-actin cytoskeleton (a defined readout of repellents/repulsive signaling). Herein, we add to this by simply driving a constitutively active form of Mical, which strongly disassembles F-actin/remodels cells in vivo independent of repulsive cues – and find that loss of Abl, which mediates growth factor signaling in these cells, decreases Mical's F-actin disassembly/cellular remodeling effects. Thus, our results are consistent with a hypothesis that cues defined as positive effectors of movement (growth factors/chemoattractants) can at least in some contexts enhance the F-actin disassembly and remodeling activity of repellents.

Dynamic changes to the actin cytoskeleton underlie the various mechanics necessary for cells to change their shape, move, and navigate [1]. Extensive studies over the past several decades have revealed that various extracellular cues regulate these actin cytoskeletal changes [2–6]. For example, signaling mediated by one of the largest families of extracellular guidance cues, Semaphorins (Semas) and their Plexin (Plex) receptors, induces actin filament (F-actin) disassembly and negatively regulates the shape, movement, and navigation of a wide range of cells and their membranous processes [7]. We have therefore chosen Semas and Plexins as a model to better understand the mechanisms by which extracellular cues regulate the actin cytoskeleton. In particular, our work has focused on deciphering how Semas/Plexins induce their effects. What are the cytoplasmic proteins involved in Sema/Plexin-mediated actin cytoskeletal rearrangements? What are the biochemical means through which they disassemble filaments? How do Semas/Plexins exert their effects to precisely specify changes in cell morphology, motility, and navigation?

One of the proteins we have found to be involved in Sema/Plexin-triggered actin rearrangements is a member of a new family of actin regulatory proteins, the MICALs, that are phylogenetically conserved both functionally and structurally from Drosophila to humans [8–11]. MICALs are large cytoplasmic proteins that contain an enzymatically active oxidation-reduction (redox) domain – which we have found uses actin filaments as a substrate and posttranslationally oxidizes the methionine 44 and 47 residues within the D-loop of actin to induce F-actin disassembly [12–14]. Our work together with the Reisler lab has also gone on to determine that the MICALs do not simply function in isolation but synergize with other well-known actin regulatory proteins such as cofilin to potentiate their effects [15]. Thus, our results present a model where Mical is activated by binding to Sema-activated Plexin to induce F-actin disassembly in a localized manner [11]. This identification of Mical and characterization of Mical's F-actin disassembly activity has thus provided a basis for better understanding the mechanisms by which extracellular cues like Semas precisely regulate the actin cytoskeleton. However, these findings have also raised a number of additional questions including: How is the activity of the MICALs precisely turned on and off? Are there other molecules that function with the MICALs in Sema/Plex signaling? Do the MICALs work with other extracellular cues? What is role of the different domains of the MICALs in their actin regulatory activity?

We have recently gained insights into some of these questions by uncovering that Mical associates with the Abl tyrosine kinase to control actin cytoskeletal dynamics in response to extracellular cues [16]. In particular, Abl is a non-receptor tyrosine kinase that plays pivotal but incompletely understood roles in actin-dependent physiological and pathological processes [17,18]. In contrast to Mical, which works with Semas-Plexins (i.e., factors that negatively affect cell movement), Abl is best known to work with growth factors and chemoattractants (i.e., factors that have been classified as having positive effects on cell movement). Therefore, we sought to define the interplay between these seemingly antagonistic effectors. Surprisingly, our results using purified proteins, site-directed mutagenesis, and enzymatic assays demonstrated that Abl directly phosphorylates Mical and *increases* Mical's repulsive effects – directly stimulating Mical's ability to dismantle actin filaments [16]. A similar Abl kinase-dependent enhancement of Mical-mediated F-actin disassembly was also observed in cellular assays in vitro and in vivo – effects that were both necessary and sufficient for cellular remodeling, axon guidance, cancer cell invasion, colony formation, and tumorigenesis [16]. Moreover, we found that Abl works in combination with growth factors to promote Semaphorin/Plexin/Mical repulsive signaling [16]. These results therefore demonstrated that a recognized “growth-promoting” effector pathway functions to magnify the F-actin disassembly and repulsive effects of a “growth-preventing” pathway (Fig. 1A [16]).

**Figure 1.**
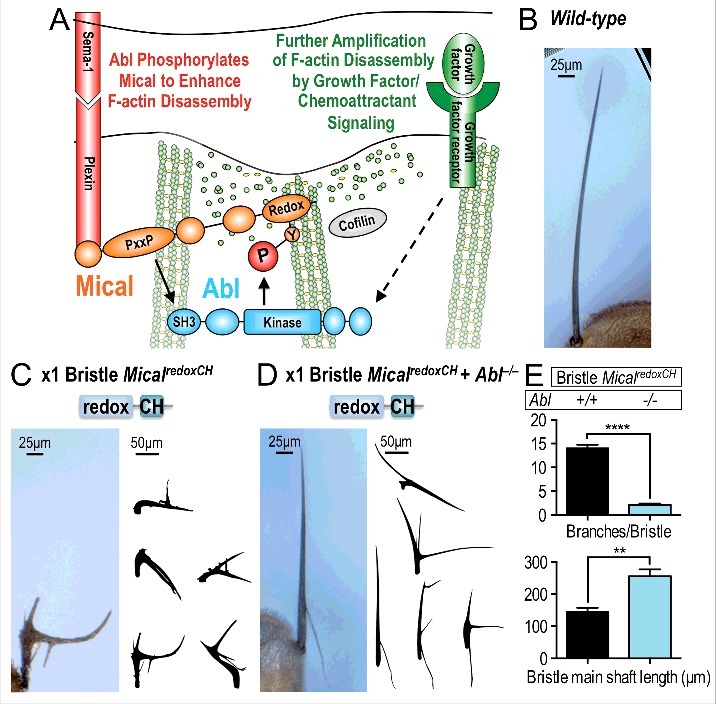
Working model and further analysis of Mical and Abl interactions. Summary working model of Mical and Abl interactions in response to Sema/Plexin and growth factor signaling. Mical is activated by Sema/Plexin repulsive guidance signaling. A PxxP motif on Mical associates with the SH3 domain of Abl. This binding is then thought to activate Abl, which phosphorylates Mical. Abl-phosphorylated Mical more actively disassembles F-actin. Growth factor signaling also activates Abl, and further amplifies Sema-Plexin-Mical F-actin disassembly and repulsion (for additional details see[16]). (B) A wild-type bristle is long, unbranched, and slightly curved. (C) Using the bristle-specific B11-GAL4 driver[12,21] to express Mical^redoxCH^ (a constitutively active form of Mical that does not contain the Plexin interaction region or the proline-rich region that mediates the interaction with the Abl SH3 domain) induces F-actin disassembly/cellular remodeling that generates stunted, branched bristles. See also[12] for more imaging of this genotype. (D) Decreasing the levels of Abl (*Abl^2^/Abl^4^* mutant alleles) suppresses Mical^redoxCH^-induced F-actin remodeling/bristle branching and restores bristle processes to a more normal length. (E) n ≥ 20 bristles/genotype; mean ± s.e.m.; t-test; ****P < 0.0001, **P < 0.01.

Our recent results therefore uncovered contexts in which recognized positive effectors of growth/guidance stimulate such negative cellular effects as F-actin disassembly/repulsion. Thus, our new results bring together these two disparate classes of cues for a common effect and provide new insights into how cells and their membranous processes are instructed to grow, and what drives them to come together or avoid one another [16,19]. Here, we have sought to continue to explore these interactions and we did this by further defining the role of the Abl kinase in regulating Mical. Our previous results had revealed that the N-terminus of Mical – containing only the Redox enzymatic domain and Calponin homology (CH) domain (what we have called Mical^redoxCH^) – is a constitutively active form of Mical that directly disassembles F-actin *in vitro* and generates severe F-actin dismantling and cellular remodeling *in vivo* [12]. Our recent work demonstrated that Abl directly phosphorylates and amplifies the F-actin disassembly activity of the Mical^redoxCH^ protein *in vitro* [16]. Thus, we wondered if Abl also affected the actin regulatory activity of Mical^redoxCH^ *in vivo*. To test this, we used the Drosophila bristle cell as a model – which is a simple high-resolution single-cell model for characterizing actin-dependent events in vivo (Fig. 1B; [20–22]). Further, both *Mical* and *Abl* are required for controlling proper bristle morphology and we used the bristle model in our recent studies defining the interactions between Mical and Abl [16]. We therefore expressed Mical^redoxCH^ in bristles in both a *wild type* background and in an *Abl* homozygous mutant background, and employed approaches for using the bristle process to look at those effects [16]. Notably, *Abl* mutants strongly suppressed the dramatic effects on F-actin/cellular remodeling that Mical^redoxCH^ induces in vivo (Fig. 1C-E, and not shown). These results thus reveal that similar to our results in vitro, Abl is also required for the full activity of constitutively-active Mical^redoxCH^ *in vivo*.

Cells and their membranous processes including nerve growth cones respond to a variety of environmental cues that control their shape, advancement, and direction of growth. These cues may be soluble or substrate bound and have historically been classified into two independently acting and antagonistic groups: growth-promoting/attractants (inducing turning toward the source of the factor ⁄ positive chemotropism) or growth-preventing/repellents (turning away from the source of the factor ⁄ negative chemotropism). Paradoxically, our recent results [16] supported by those herein find a previously unknown role for chemoattractant cues in promoting the effects of repellent cues and thereby amend conventional views regarding the antagonistic action of these opposing groups of cues. In particular, since our previous work [16] directly links the action of growth factors/chemoattractants and their signaling pathways to the promotion of the disassembly of the F-actin cytoskeleton (a defined readout of repellents/repulsive signaling), our results contradict these simple groupings by revealing that “attractants” can serve to promote the negative effects of “repellents”. Herein, we add to this by simply driving a constitutively active form of Mical, which directly disassembles F-actin independent of Semas/Plexins [12] – and find that loss of Abl (which we have found previously to mediate growth factor signaling in these cells [16]) decreases Mical's F-actin/cellular remodeling effects. Thus, our results are consistent with a hypothesis that cues defined as positive effectors of movement (growth factors/chemoattractants) can at least in some contexts enhance the F-actin disassembly activity of repellents.

In light of our work, it is interesting that previous studies have also suggested occasions where known growth factor/attractants can be linked to known repellents. For example, growth factor receptors have often been found in association with Semas/Plexins (noted in [7,16]) – including examples where they can be found to assist one another, although this has not been categorized as repulsion. Further, although in some cases these interactions are not mediated through classical growth factor receptors, observations from other labs with growth factors/chemoattractants are consistent with them having a role in increasing F-actin disassembly/repulsion (e.g. [23–29]). Thus, the classification of extrinsic cues into one of two groups is a simplification that likely misrepresents the direct roles of specific cues. Indeed, these extracellular signals and their positive or negative effects have often been defined solely on the basis of complex in vitro and in vivo cellular assays whereby outcomes may be indirect and may obscure the direct roles of specific cues. Thus, more work needs to be done to understand the actions of these cues – including their effects when presented in combination, context-dependent differences in their effects, and systematically defining the intracellular signaling pathways utilized by each of these extrinsic cues. It is our strategy that coupling clear single-cell in vivo genetic and cellular models with sensitive biochemical assays using purified proteins will help resolve the incoherent picture by which a multitude of extracellular cues combine together to direct both cells and their membranous processes to their final destination.

## Materials and methods

Analysis of bristle cells for morphological defects was done as described previously [12,16] by mating flies at 25°C and subjecting recently emerged adult offspring to collection, genotyping, and qualitative and quantitative analysis under a dissecting microscope (Leica Stereo Zoom S8 APO). Bristles were examined for defects in morphology including branching as described previously [12,16]. The number of branches on each bristle was counted and the results were presented as the mean number of branches per bristle (± the standard error of the mean (SEM)). Bristle imaging and drawings were done with the aid of a Zeiss Discovery M2 Bio stereomicroscope, a Zeiss Axiocam HR camera, a motorized focus and zoom, three-dimensional reconstruction software (Zeiss Axiovision software and Extended Focus Software [a kind gift from Bernard Lee]), and Microsoft Office Powerpoint, as described [12,16]. The bristle specific *B11-GAL4* driver, the Mical^redoxCH^ transgenic line (*UAS:Mical^redoxCH^*), and the *Abl* mutant lines were as previously described (see [12,14,16]).
